# Extracellular vesicles in cancer-associated cachexia: molecular mechanisms and therapeutic perspectives

**DOI:** 10.3389/fcell.2026.1929088

**Published:** 2026-09-11

**Authors:** An Xie, Shuzhou Wu, Wenhui Zeng, Mengting Zhang, Jiabing Wang, Lei Shi, Nan Wu, Bingli Hu, Junsong Ye, Zhengwei Zou, Lincai Li, Lin Zhou

**Affiliations:** 1 Stem Cell Clinical Translation Center, First Affiliated Hospital of Gannan Medical University, Ganzhou, Jiangxi, China; 2 School of Rehabilitation and Exercise Health Medicine, Gannan Medical University, Ganzhou, Jiangxi, China; 3 School of Pharmacy, Gannan Medical University, Ganzhou, Jiangxi, China; 4 School of Medical Technology Gannan Medical University, Ganzhou, Jiangxi, China; 5 The First Clinical College of Gannan Medical University, Ganzhou, Jiangxi, China; 6 Ganzhou Key Laboratory of Stem Cell and Regenerative Medicine, Ganzhou, Jiangxi, China; 7 Key Laboratory of Prevention and Treatment of Cardiovascular and Cerebrovascular Diseases, Ministry of Education, Gannan Medical University, Ganzhou, Jiangxi, China; 8 Key Laboratory for Tissue Engineering of Jiangxi Province, Gannan Medical University, Ganzhou, Jiangxi, China

**Keywords:** biomarkers, CAC, EVs, inter-organ communication, skeletal muscle atrophy, therapeutic strategy

## Abstract

Cancer-associated cachexia (CAC) is increasingly recognized as a systemic metabolic disorder characterized by progressive skeletal muscle wasting, adipose tissue remodeling, chronic inflammation, and profound metabolic dysfunction, which collectively compromise therapeutic efficacy and patient survival. Although accumulating experimental studies have demonstrated that tumor-derived extracellular vesicles (EVs) contribute to CAC, current evidence is largely fragmented, with most studies focusing on individual EV cargoes or isolated signaling pathways rather than providing an integrated mechanistic perspective. This review evaluates representative experimental studies describing how tumor-derived EVs regulate skeletal muscle atrophy, adipose tissue remodeling, inflammatory responses, and metabolic reprogramming during cachexia progression. We further summarize recent advances in EV-based biomarkers and therapeutic strategies, while discussing current challenges and future directions for clinical translation. In particular, circulating EV-associated molecules, including glucose-regulated protein 75 (GRP75) and specific miRNAs, have shown promise as diagnostic and prognostic biomarkers. Unlike previous reviews that primarily focus on individual EV cargoes or signaling pathways, this review integrates representative experimental evidence spanning molecular mechanisms, biomarker discovery, and therapeutic intervention, thereby providing a comprehensive overview of the multifaceted roles of tumor-derived EVs in CAC and their translational potential as biomarkers and therapeutic targets.

## 1 Introduction

CAC is a multifaceted metabolic disorder characterized by progressive skeletal muscle loss, with or without concomitant adipose tissue loss, and is frequently accompanied by systemic inflammation (Baracos et al., 2018). CAC affects approximately 50%–80% of patients with advanced malignancies and is particularly prevalent in pancreatic and gastric cancers (Shibata et al., 2020; White et al., 2020). Notably, conventional nutritional support is insufficient to reverse cachexia progression, and severe weight loss is strongly associated with increased mortality (Busquets et al., 2007; Arends et al., 2021; Hussain et al., 2023). In addition, CAC markedly impairs physical function and overall quality of life (Vagnildhaug et al., 2017). Current clinical management primarily relies on nutritional support and symptomatic interventions, which fail to reverse the underlying metabolic and catabolic alterations in skeletal muscle and adipose tissue (Vagnildhaug et al., 2017; Wang Y. et al., 2024). Therefore, a more comprehensive understanding of the molecular mechanisms underlying CAC is essential to develop effective therapeutic strategies.

The tumor-derived secretome refers to the collection of bioactive molecules secreted by tumor cells into the extracellular microenvironment and systemic circulation, including soluble proteins, peptides, nucleic acids, and EVs (Gurung et al., 2021; Swietlik et al., 2023). Studies have shown that the tumor secretome serves as a key mediator of tumor-host communication, playing a central role in regulating skeletal muscle metabolism and promoting CAC progression (Zhang et al., 2022). Although early studies primarily focused on soluble circulating factors, soluble factors alone are insufficient to explain the systemic and coordinated metabolic remodeling observed in CAC. These observations suggest that additional mechanisms of intercellular communication may be involved. EVs, including exosomes, act as key regulators of tumor-host crosstalk through the intercellular delivery of proteins, lipids, and nucleic acids.

Compared with classical soluble factors, EVs provide a more stable and coordinated mode of signal delivery, allowing simultaneous modulation of multiple metabolic pathways (Thakur et al., 2014). Notably, circulating levels of specific exosomal miRNAs and proteins have been inversely correlated with skeletal muscle mass and overall survival (Yamada et al., 2015), positioning them as promising non-invasive indicators for early CAC diagnosis. However, a comprehensive framework integrating EV cargo heterogeneity, inter-organ communication, and metabolic reprogramming in CAC is still lacking. Therefore, this review summarizes current evidence supporting EV-mediated tumor-host communication as a key mechanism underlying the initiation and progression of CAC, with particular emphasis on its biological functions, clinical implications, and therapeutic potential.

## 2 Classical tumor-derived circulating factors regulating CAC

**FIGURE 1:**
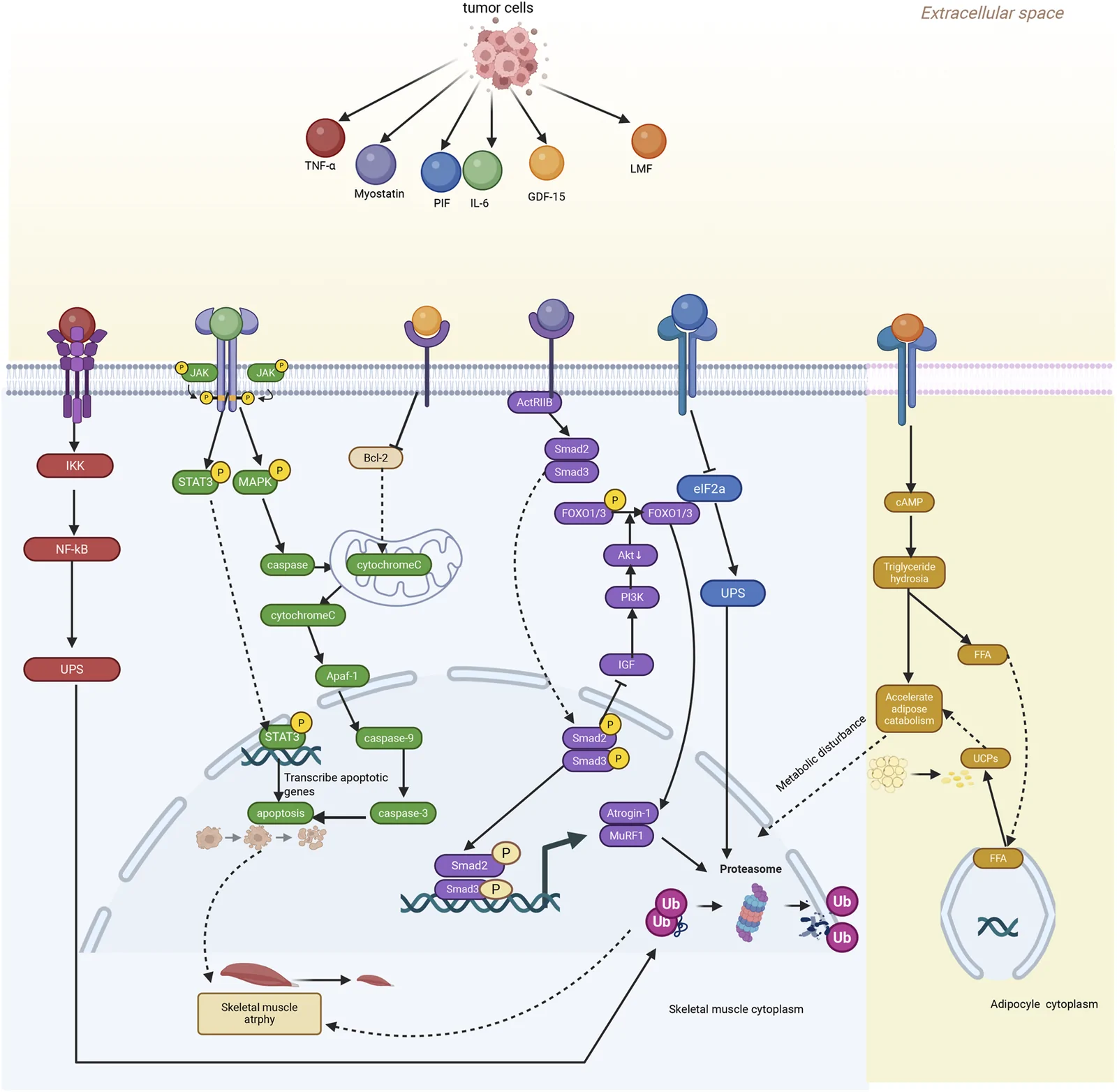
Classic circulating factors induce CAC (including TNF-α, IL6, MSTN, PIF, GDF15, and LMF). 1. TNF-α induces NF-κB activation via IKK-mediated phosphorylation and degradation of IκB. 2. IL-6 activates the JAK-STAT3 and MAPK pathways. 3. GDF15 activates the *Bcl-2*/caspase-3 pathway. 4. MSTN activates Smad2/3 and inhibits the AKT/mTOR pathway. 5. PIF Inhibits eIF2α and activates the ubiquitin-proteasome pathway. 6. LMF promotes cAMP-mediated triglyceride hydrolysis and upregulates UCP. Collectively, these factors drive CAC through multiple mechanisms, culminating in the induction of skeletal muscle atrophy and lipolysis. Created in BioRender. an, X. (2026) https://BioRender.com/slvnrxo.

Classical circulating factors contribute to CAC primarily through inflammatory signaling, muscle proteolysis, and systemic metabolic remodeling, as illustrated in Figure 1.

### 2.1 Core factors and functions

#### 2.1.1 Inflammation-driven signaling

Pro-inflammatory cytokines, particularly TNF-α and IL-6, are central mediators of systemic inflammation in CAC. These cytokines promote skeletal muscle wasting and metabolic dysfunction through activation of inflammatory and catabolic signaling pathways, including NF-κB and JAK/STAT3 pathways (Miller et al., 2017; Arora et al., 2020; Yi et al., 2022). Additionally, anti-inflammatory cytokines, such as IL-10, may also contribute to cachexia progression in specific tumor contexts by modulating immune-metabolic interactions (Robert et al., 2012; Li et al., 2016). Collectively, these cytokines contribute to muscle atrophy and cachexia progression through inflammatory and immune-metabolic signaling pathways.

#### 2.1.2 Muscle catabolic factors

Beyond pro-inflammatory cytokines, several tumor-derived factors directly regulate muscle catabolism. Pro-cachectic factors such as myostatin (MSTN) and proteolysis-inducing factor (PIF) play critical roles in muscle catabolism (Kim-Muller et al., 2023). MSTN, a member of the TGF-β superfamily, suppresses muscle growth through activation of Smad-dependent catabolic signaling and inhibition of the anabolic IGF-1/PI3K/Akt pathway, thereby promoting activation of proteolytic programs (Lokireddy et al., 2011; Goebel et al., 2022; Salemi et al., 2023). Simultaneously, MSTN suppresses the anabolic IGF-1/PI3K/Akt pathway, resulting in reduced Akt phosphorylation and subsequent nuclear accumulation of FOXO transcription factors (FOXO1/FOXO3), which further amplify proteolytic signaling (Goebel et al., 2022; Lee and McPherron, 2001).

PIF is produced by cachectic tumors and has been detected in patients with pancreatic cancer and non-small cell lung cancer (NSCLC), with higher levels associated with poor clinical outcomes (Wigmore et al., 2000; Wang et al., 2010). Mechanistically, PIF promotes skeletal muscle wasting by enhancing protein degradation and suppressing protein synthesis (Tisdale, 2010). Collectively, these factors converge on common catabolic signaling pathways, leading to progressive muscle loss in CAC.

#### 2.1.3 Metabolic regulators

Systemic metabolic regulators, including growth differentiation factor 15 (GDF-15) and lipid-mobilizing factor (LMF), contribute to the multi-organ metabolic alterations observed in CAC. GDF-15, a distant member of the TGF-β superfamily, was originally identified as macrophage inhibitory cytokine-1 (Wang L. et al., 2024). GDF-15 induces C2C12 myotube atrophy by downregulating *Bcl-2* and activating caspase-3, thereby promoting apoptosis (Zhang et al., 2022). LMF is a zinc-α2-glycoprotein that stimulates triglyceride hydrolysis via the cAMP signaling pathway, leading to white adipose tissue (WAT) loss. Moreover, LMF enhances fatty acid oxidation and thermogenesis through the upregulation of uncoupling proteins (UCPs) (Bing et al., 2002; Sanders and Tisdale, 2004). Rather than indicating direct catabolism of brown adipose tissue (BAT), LMF-induced UCP activation promotes mitochondrial uncoupling and increases energy expenditure. In CAC, this thermogenic program, together with enhanced lipolysis and depletion of WAT lipid stores, contributes to systemic energy imbalance and progressive adipose tissue wasting. The key circulating factors and their biological functions are summarized in Table 1.

**TABLE 1:** Classic circulating factors induce skeletal muscle atrophy and lipolysis.

Circulating factors	Target genes/pathways	Target cell types	Biological functions	References
TNF-α	IκB	skeletal muscle cells	Activation of IκB and NF-κB, initiating the ubiquitin-proteasome degradation pathway → Acceleration of skeletal muscle catabolic metabolism	Patel and Patel (2017)
IL-6	JAK	skeletal muscle cells	Activation of the JAK-STAT3 and MAPK pathways → Induction of skeletal muscle cell apoptosis	Miller et al. (2017); Johnson et al. (2018)
PIF	eIF2α	skeletal muscle cells	Inhibition of eIF2α and activation of the ubiquitin-proteasome pathway → Inhibition of skeletal muscle protein synthesis and acceleration of its degradation	Tisdale (2010)
GDF15	*Bcl-2*	C2C12 myotubes	Activation of the *Bcl-2*/caspase-3 pathway → Induction of skeletal muscle cell apoptosis	Zhang et al. (2022)
LMF	UCP	adipocyte	Induction of cAMP-mediated triglyceride hydrolysis and elevation of UCP → Enhancement of lipolysis and its conversion to thermal energy	Bing et al. (2002)
MSTN	Smad, Akt	skeletal muscle cells	Binding to and activation of the ActRIIB receptor, activation of Smad2/3, and inhibition of the AKT/mTOR pathway → Promotion of skeletal muscle protein degradation	Lokireddy et al. (2011); Lee and McPherron (2001)

### 2.2 Regulatory pathways and biological effects

Classical tumor-derived circulating factors disrupt systemic homeostasis through several interconnected mechanisms, including enhanced protein degradation, impaired protein synthesis, inflammation, and apoptosis. These factors activate catabolic pathways such as the UPS and autophagy while suppressing anabolic signaling pathways, including mTOR and PI3K/Akt signaling, ultimately leading to skeletal muscle wasting and metabolic dysfunction (Pijet et al., 2013; De Larichaud et al., 2012; Onesti and Guttridge, 2014; Hu F. et al., 2021; Petruzzelli and Wagner, 2016). Collectively, these mechanisms demonstrate that CAC results from coordinated alterations in multiple metabolic processes rather than a single pathogenic pathway.

### 2.3 Limitations of single-factor models in CAC

Despite extensive investigation, therapies targeting individual circulating factors have shown limited clinical efficacy. For example, anti-TNF-α therapies failed to effectively reverse skeletal muscle loss in patients with CAC (Jatoi et al., 2010; Monk et al., 2006). These findings support the multifactorial nature of CAC and highlight the limitations of the single-factor paradigm. In this context, EVs, which coordinate the transfer of multiple bioactive cargoes, provide a complementary framework for understanding the complex tumor–host communication underlying CAC.

## 3 The regulatory role of tumor-derived EVs in CAC

Tumor-derived EVs act as critical mediators in CAC, operating through their biological classification, unique regulatory properties, and integrated nucleic acid-, epigenetic-, and protein-dependent mechanisms.

### 3.1 Biological classification of EVs

EVs, especially exosomes, are now regarded as important vehicles of intercellular communication in cancer and are increasingly implicated in the development of CAC (Liu Y. et al., 2022). EVs constitute a heterogeneous population of membrane-bound vesicles derived from cells, either released through plasma membrane budding or formed via the endosomal pathway (van Niel et al., 2018; Bebelman et al., 2018). They carry diverse bioactive molecules, including DNA, coding and non-coding RNAs (ncRNAs, e.g., miRNA, lncRNA, circRNA), proteins (e.g., transmembrane proteins CD9, CD63, and HSPs), lipids, glycostructures, and metabolites (Théry et al., 2018). EVs mediate intercellular communication by transporting bioactive molecules within their lumen or on their surface (Nikonorova et al., 2022), thereby regulating physiological and pathological processes (Yang et al., 2020; Willms et al., 2016). The biogenesis and secretion of EVs are tightly regulated by intracellular signaling pathways. For instance, STAT3 is recognized as a central regulator involved in the biogenesis of EVs in tumor cells, linking inflammatory signaling with EV-mediated communication in CAC (Fan et al., 2022a). EVs are generally classified into three major subtypes, namely, exosomes (originating from the endosomal system, 30–150 nm in diameter), ectosomes/microvesicles (formed by direct budding from the plasma membrane, 100–1000 nm (Cocucci et al., 2009; Kowal et al., 2016)), and apoptotic bodies (50–5000 nm). Among the various extracellular vesicle populations, small EVs can be operationally enriched using methods based on physical properties such as size and density, including ultracentrifugation and density-based separation. However, according to current ISEV guidelines, no available method can selectively isolate a specific EV subpopulation with complete specificity, and small EVs should not be considered synonymous with exosomes (Willms et al., 2016; Kong et al., 2026; Welsh et al., 2024). The choice of isolation method can also affect EV purity, integrity, and functional properties, with polymer-based precipitation methods being particularly prone to co-isolating non-EV components and altering EV characteristics (Welsh et al., 2024; Paolini et al., 2016). Therefore, standardized and well-characterized EV isolation strategies remain important for improving reproducibility. EVs possess several unique biological properties that distinguish them from classical soluble factors and underpin their regulatory roles in CAC. First, EVs function as protected carriers. Their lipid bilayer structure shields encapsulated cargo from degradation by nucleases and proteases, thereby prolonging their stability and bioavailability in circulation (Kong et al., 2026). Second, EVs exhibit intrinsic targeting capability. Surface molecules such as integrins and receptor-associated proteins facilitate selective interactions with recipient cells, enabling efficient cargo delivery (Chen et al., 2024a; Hoshino et al., 2015). In contrast, soluble circulating factors lack a protective lipid bilayer and undergo extensive diffusion in the bloodstream, resulting in reduced delivery efficiency and a shorter duration of action. Third, EVs enable coordinated multi-molecule regulation. A single EV can simultaneously deliver diverse bioactive components, allowing for synergistic modulation of multiple signaling pathways. For example, EVs derived from colon cancer cells have been shown to co-deliver miR-195a-5p and miR-125b-1-3p, which suppress *Bcl-2* expression and promote apoptotic signaling in skeletal muscle cells (Miao et al., 2021).

### 3.2 Primary mechanisms of EV-mediated regulation of skeletal muscle

**FIGURE 2:**
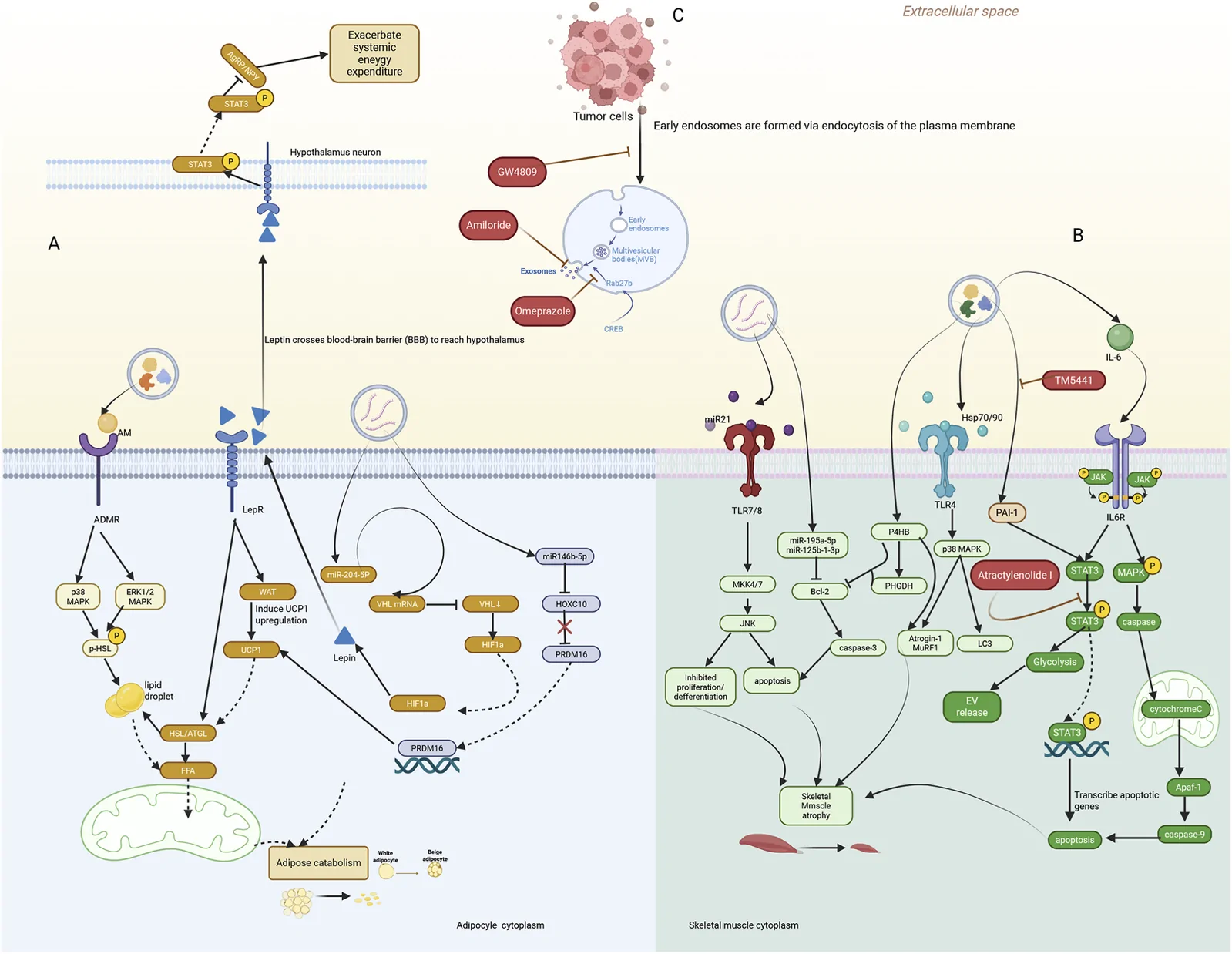
Mechanisms of tumor-derived EVs mediated CAC and targeted pharmacological interventions. Tumor-derived exosomes deliver miRNAs, proteins, and cytokines to drive adipose lipolysis and skeletal muscle atrophy in cancer cachexia via distinct signaling pathways. **(A)** Exosomal mediators of adipose lipolysis. 1. Pancreatic cancer exosomes: AM activates p38/ERK1/2 MAPK signaling. 2. Breast cancer exosomes: miR-204-5p inhibits *VHL* and stabilizes HIF-1α. 3. Colorectal cancer exosomes: miR-146b-5p relieves *HOXC10*-mediated PRDM repression and upregulates PRDM. **(B)** Exosomal inducers of skeletal muscle atrophy/apoptosis. 1. Lung and pancreatic cancer exosomes: miR-21 activates the JNK pathway. 2. Colorectal cancer exosomes: miR-195a-5p/miR-125b-1-3p suppresses *Bcl-2* expression. 3. ESCC exosomes: P4HB modulates PHGDH/*Bcl-2*/caspase-3 pathway. 4. LLC exosomes: HSP70/90 activates p38 MAPK signaling. 5. Glioblastoma exosomes: P4HB activates STAT3 pathway. 6. C26 colon carcinoma exosomes: IL-6 inhibits STAT3/PKM2/SNAP23 signaling pathway. **(C)** Pharmacological interventions for exosome-driven CAC. 1. Atractylenolide I: Inhibits STAT3/PKM2/SNAP23 pathway; reduces exosome biogenesis and IL-6 secretion. 2. GW4869: Blocks biogenesis and release of tumor-derived exosomes. 3. Amiloride: Impairs exocytic release of MVBs. 4. Omeprazole: Downregulates Rab27b expression and impairs MVB maturation. 5. TM5441 (PAI-1 inhibitor): Blocks the STAT3 signaling pathway activated by exosomal PAI-1. Created in BioRender. an, X. (2026) https://BioRender.com/slvnrxo.

The primary mechanisms underlying EV-mediated regulation of skeletal muscle are broadly classified into four categories, as illustrated in Figure 2.

#### 3.2.1 Nucleic acid-mediated regulation

EV-derived miRNAs constitute one of the principal mechanisms driving skeletal muscle atrophy in CAC. Among these, miR-21 has been extensively characterized. Tumor-derived EVs carrying miR-21 activate JNK signaling via TLR7 (in mice) or TLR8 (in humans), leading to apoptosis and impaired myogenic differentiation (He et al., 2014). In addition, miRNAs such as miR-195a-5p and miR-125b-1-3p directly suppress anti-apoptotic genes including *Bcl-2*, further exacerbating muscle atrophy (Miao et al., 2021). Similar observations have also been reported in EVs released by cancer-associated fibroblasts, which deliver miR-125b and contribute to skeletal muscle atrophy (Kim et al., 2026). Similarly, EVs derived from oral squamous cell carcinoma (OSCC) cells carry miR-181a-3p, which modulates endoplasmic reticulum stress and UPS activity, thereby promoting muscle atrophy and apoptosis (Qiu et al., 2020). These findings highlight two distinct but complementary modes of action, namely, receptor-mediated activation of intracellular signaling pathways and post-transcriptional regulation following EV internalization. Together, these mechanisms enable EV-associated miRNAs to regulate both upstream signaling pathways and downstream apoptotic responses.

However, several critical questions remain unresolved. The mechanisms governing selective miRNA loading into EVs are not fully understood. In addition, the heterogeneous origins of EVs from multiple cell types complicate the identification of tumor-specific signals. Improving targeting specificity and validating these findings in clinical settings will be essential for future translation. The cargoes and biological functions of tumor-derived EVs are summarized in Table 2.

**TABLE 2:** The cargoes, including ncRNAs and proteins, secreted by tumor-derived exosomes drive skeletal muscle atrophy and lipolysis.

Cancer type	EV cargo	EV source	Recipient tissue	Experimental model	Biological functions	Evidence level	References
Pancreatic and lung malignancies	miR21	Tumor-derived EVs	Skeletal muscle	C2C12 myoblasts + tumor-bearing mice	Activation of TLR7 receptor on murine myoblasts → Increasing JNK activity promotes myocyte apoptosis	*In vitro* + *in vivo*	He et al. (2014)
C26 colorectal cancer	miR-195a-5p and miR-125b-1-3p	C26-derived exosomes	Skeletal muscle	C2C12 myotubes + C26 tumor-bearing mice	Inhibition of the *BCL2* gene leads to *BCL2* downregulation → Activates the apoptotic signaling pathway	*In vitro* + *in vivo*	Miao et al. (2021)
Oral squamous cell carcinoma	miR-181a-3p	OSCC-derived exosomes	Skeletal muscle	C2C12 myotubes + SCC7 xenograft mice	Regulation of the ERS and UPS pathways → Inducing skeletal muscle atrophy and apoptosis	*In vitro* + *in vivo*	Qiu et al. (2020)
Lung cancer	Hsp70/90	LLC-derived EVs	Skeletal muscle	C2C12 myotubes + LLC tumor-bearing mice	Activation of the p38 MAPK pathway → Upregulation of the E3 ubiquitin ligases Atrogin-1. Induces the activation of the autophagy marker LC3 → Enhancing autophagy in skeletal muscle cells	*In vitro* + *in vivo*	Zhang et al. (2017b)
Glioblastoma (GBM)	PAI-1	GBM-derived exosomes	Skeletal muscle	Radiation-treated GBM cells + C2C12 myotubes + GBM xenograft mice	Activation of the STAT3 pathway → Upregulates the gene expression of MuRF-1 and Atrogin-1. Inhibits the activation of the mTOR signaling pathway → Suppressing skeletal muscle protein synthesis	*In vitro* + *in vivo*	Shin et al. (2022)
Esophageal squamous cell carcinoma	P4HB	ESCC-derived EVs	Skeletal muscle cells	C2C12 myotubes + ESCC xenograft mice	Regulation of the PHGDH/*Bcl-2*/caspase-3 pathway → Induce skeletal muscle atrophy	*In vitro* + *in vivo*	Gao et al. (2021)
Breast cancer	miR-204-5p	Breast cancer-derived small extracellular vesicles (sEVs)	Adipose tissue	Primary adipocytes + MDA-MB-231 tumor-bearing mice	Inhibition of the *VHL* gene enhances the stability of HIF-1α protein → Activation of the leptin signaling pathway promotes the browning of WAT and lipolysis	*In vitro* + *in vivo*	Hu et al. (2023)
Colorectal cancer	miR-146b-5p	Colorectal cancer-derived exosomes	Adipose tissue	Adipocytes + colorectal cancer tumor-bearing mice	Suppression of *HOXC10* → upregulation of browning-related genes (e.g., PRDM16 and UCP1) → WAT browning and enhanced lipolysis	*In vitro* + *in vivo*	Di et al. (2021)
Pancreatic cancer	AM	Pancreatic cancer-derived exosomes	Adipose tissue	3T3-L1 adipocytes + human subcutaneous adipocytes	Binding to ADMR activates the downstream P38 and ERK1/2 MAPK signaling pathways → Triggers lipolysis in adipocytes	*In vitro*	Sagar et al. (2016)
Lung cancer/Colorectal cancer	IL-8	LLC- and C26-derived EVs	Adipose tissue Adipose tissue	3T3-L1 adipocytes + LLC/C26 tumor-bearing mice	IL-8/CXCR2 interaction → NF-κB activation → increased PGC1α/UCP1 expression → adipocyte lipolysis and wasting	*In vitro* + *in vivo*	Xiong et al. (2022)

#### 3.2.2 Epigenetic and transcriptional reprogramming

Beyond directly activating catabolic pathways, EVs also regulate skeletal muscle metabolism through epigenetic and transcriptional reprogramming. For example, EVs enriched in miR-223-5p suppress the transcription factor MAFA, leading to downregulation of the m6A methyltransferase METTL14 and a global reduction in RNA methylation (Xu et al., 2025). In addition, integrated multi-omics analyses have linked EV-associated miRNAs, such as miR-29a-3p, to alterations in amino acid metabolism, particularly alanine, aspartate, and glutamate metabolism (Bafiti et al., 2024).

These findings reveal a previously unappreciated mechanism by which EV-derived miRNAs mediate crosstalk between tumors and skeletal muscle, operating through the modulation of RNA epigenetic modifications. This mechanism provides new insights into the multifaceted pathophysiology underlying CAC. EV-mediated regulation encompasses not only classical signaling pathways but also epitranscriptomic remodeling. Despite these advances, evidence remains limited.

#### 3.2.3 Protein-mediated regulation

In addition to EV-derived RNAs, EV-associated proteins constitute another major class of mediators in CAC. Hsp70/90 activate the TLR4-p38 MAPK signaling pathway, leading to the upregulation of Atrogin-1 and MuRF-1 while simultaneously inducing autophagy (Zhang et al., 2017a). Upstream regulators such as ZIP4 further enhance this process by promoting EV secretion via RAB27b-dependent mechanisms (Yang et al., 2019). Additional EV-associated proteins, plasminogen activator inhibitor-1 (PAI-1) and prolyl 4-hydroxylase subunit beta (P4HB), induce skeletal muscle atrophy through STAT3 activation and apoptosis-related pathways (Shin et al., 2022; Naderi et al., 2009; Chen et al., 2024b). Beyond their effects on skeletal muscle, EV-associated proteins also modulate systemic metabolism. GRP75 promotes mitochondrial uncoupling and thermogenesis in adipose tissue by stabilizing ANT2 and enhancing UCP1 expression, thereby contributing to systemic energy imbalance (Chen et al., 2024b; Zhang et al., 2021). Despite targeting different upstream processes, these proteins converge on common downstream pathways, including the UPS, autophagy, and apoptosis.

However, several key questions remain unresolved. The mechanisms governing selective protein loading into EVs are poorly understood, and the relative contribution of EV-mediated versus soluble protein signaling requires further clarification. In addition, most current evidence remains correlative, highlighting the need for validation in clinically relevant models. Further validation in clinically relevant models is required before clinical translation can be achieved.

#### 3.2.4 Synergistic regulation with other circulating factors

In addition to their independent effects, EVs synergize with classical tumor-derived circulating factors to amplify CAC progression. For example, EVs cooperate with IL-6 to activate STAT3 and NF-κB signaling pathways, leading to skeletal muscle atrophy and adipose tissue wasting (Hu et al., 2019).

Interestingly, not all EVs exert detrimental effects. EVs derived from non-tumor cells, such as mesenchymal stem cells, may attenuate CAC progression. For instance, EV-associated miR-145-5p targets activin A receptor-mediated signaling and alleviates muscle atrophy *in vivo* (Cho et al., 2021). Similarly, bone marrow-derived EVs inhibit muscle atrophy through miR-486-5p-mediated suppression of FOXO1 signaling (Li Z. et al., 2021). In addition, bone marrow mesenchymal stem cell-derived exosomes (BMSC-Exos) inhibit the invasion, migration, and epithelial-mesenchymal transition (EMT) of NSCLC cells via exosomal miR-204-mediated suppression of the KLF7/AKT/HIF1α axis (Liu et al., 2021). These findings further suggest that therapeutic EVs may simultaneously suppress tumor progression and alleviate CAC.

### 3.3 EVs mediate lipolysis and WAT browning

Adipose tissue is an important metabolic target of tumor-derived EVs during CAC, undergoing extensive remodeling that contributes to systemic energy imbalance and progressive fat loss. Increasing experimental evidence indicates that tumor-derived EVs can alter adipocyte metabolism through diverse molecular cargoes and surface-associated determinants, thereby promoting lipid mobilization and WAT browning. These effects are mediated by multiple classes of EV cargoes, including miRNAs and proteins, as well as EV-associated molecular features that influence interactions with adipocytes. Recent evidence further suggests that EV-mediated adipose remodeling may participate in a bidirectional metabolic exchange between tumors and adipose tissue. More recently, Ramos et al. demonstrated that tumor-associated glycosylation of EVs can further regulate tumor-adipocyte metabolic crosstalk. STn-enriched gastric cancer-derived EVs exhibited enhanced uptake by adipocytes and induced WAT browning and lipolysis, resulting in increased release of fatty acids that were subsequently utilized by tumor cells to support fatty acid oxidation and metabolic adaptation (R et al., 2026). Thus, EV-mediated adipose tissue remodeling involves interconnected processes of enhanced lipolysis and WAT browning, which not only accelerate the depletion of adipose energy stores but may also provide metabolic substrates that support tumor adaptation. These two major mechanisms are discussed below.

#### 3.3.1 EV-mediated lipolysis

Tumor-derived EVs promote adipocyte lipolysis through multiple molecular mechanisms involving both EV cargoes and EV surface molecules. Early experimental evidence showed that pancreatic cancer-derived exosomes induce lipolysis in murine and human adipocytes, with exosomal adrenomedullin (AM) activating p38 and ERK1/2 MAPK signaling and promoting HSL phosphorylation (Sagar et al., 2016). Similarly, LLC-derived EVs can induce lipolysis in adipocytes by delivering PTHrP, which activates PTHR/PKA signaling; neutralization of PTHrP, PTHR knockdown, or inhibition of EV release markedly attenuated this effect (Hu W. et al., 2021). Subsequent studies have identified additional EV-associated mediators that regulate adipocyte lipid mobilization. EV-associated IL-8 derived from LLC and C26 cells promotes adipocyte lipolysis through CXCR2-dependent activation of NF-κB, accompanied by increased expression of PGC-1α and UCP1 (Xiong et al., 2022). More recent studies have further expanded the spectrum of EV-associated regulators involved in adipocyte lipolysis. Exosomal EIF5A derived from LLC cells promotes lipolysis in 3T3-L1 and HIB1B adipocytes by binding GPBAR1 mRNA, enhancing its translation, and activating CREB signaling; suppression of EIF5A attenuated adipose tissue lipolysis in cachectic mice (Xiong et al., 2023). In addition, pancreatic cancer-derived exosomal miR-29a-3p was shown to promote lipolysis by suppressing MCT1 and increasing ATGL expression, while depletion of miR-16-5p and miR-29a-3p from pancreatic cancer exosomes impaired their ability to induce fat loss (Tien et al., 2024).

Collectively, these findings indicate that tumor-derived EVs promote adipocyte lipolysis through multiple, mechanistically distinct pathways involving EV-associated proteins and miRNAs. This EV-mediated lipid mobilization contributes to progressive adipose tissue depletion and represents an important component of the metabolic remodeling characteristic of CAC.

#### 3.3.2 EV-mediated WAT browning

Several tumor-derived EV cargoes have been shown to promote the browning of WAT by reprogramming adipocyte differentiation (Hu et al., 2023). Colorectal cancer-derived exosomal miR-146b-5p promotes adipocyte reprogramming through the HOXC10/PRDM16 axis, favoring the acquisition of a beige adipocyte phenotype (Di et al., 2021). In gastric cancer, exosomal miR-155 has been shown to target C/EBPβ in adipose mesenchymal stem cells, resulting in reduced expression of the adipogenic regulators C/EBPα and PPARγ and increased UCP1 expression, thereby promoting brown adipose differentiation and contributing to adipose tissue loss in CAC (Liu Y. et al., 2022). Beyond miRNA cargoes, tumor-derived EV-associated GRP75 promotes WAT browning through the ANT2/UCP1 axis, while pharmacological inhibition of GRP75 with withanone attenuates EV-induced browning and alleviates cachectic phenotypes *in vivo* (Chen et al., 2024b).

Collectively, these findings indicate that tumor-derived EVs promote WAT browning through diverse molecular mechanisms involving both regulatory miRNAs and EV-associated surface features. This browning of WAT, together with EV-mediated lipolysis, may increase energy expenditure and contribute to progressive adipose tissue depletion and systemic metabolic imbalance in CAC.

### 3.4 EV-mediated systemic inter-organ communication in CAC

Beyond skeletal muscle and adipose tissue, tumor-derived EVs have emerged as key mediators of systemic inter-organ communication. By transferring diverse bioactive cargoes to distant organs, EVs coordinate neuroendocrine regulation, immune responses, and metabolic adaptation, thereby contributing to the systemic metabolic remodeling characteristic of CAC.

#### 3.4.1 EV-mediated neural and immune regulation in CAC

Tumor-derived EVs may contribute to CAC not only through direct effects on skeletal muscle and adipose tissue but also by modulating neural and immune functions. In the nervous system, EVs derived from murine breast cancer cells have been shown to induce anxiety- and depression-like behaviors, with 4T1-derived EVs further reported to promote depression-like behaviors through MYD88 upregulation (Han et al., 2026; Zhou et al., 2026). Although these studies did not directly investigate CAC, tumor-EV-mediated alterations in CNS function may affect appetite, neuroendocrine regulation, and systemic energy balance, potentially contributing to cachexia-associated metabolic disturbances.

Tumor-derived EVs can also reshape immune responses. EV-associated PD-L1 promotes CD8^+^ T-cell exhaustion, while EV-derived miRNAs, including miR-21-5p and miR-200a, promote immunosuppressive macrophage polarization and enhance PD-L1 expression in tumor-associated macrophages (Poggio et al., 2019; Chen et al., 2018; Yin et al., 2022). These immune alterations may contribute to persistent systemic inflammation, which is closely associated with skeletal muscle wasting and adipose tissue remodeling in CAC. Together, these findings suggest that EV-mediated modulation of neural and immune functions may represent additional pathways linking tumor-derived signals to the systemic metabolic disturbances of CAC, although their direct contribution to cachexia requires further investigation.

#### 3.4.2 EV-mediated hepatic and metabolic regulation in CAC

Tumor-derived extracellular vesicle and particles (EVPs) enriched in saturated fatty acids, particularly palmitic acid, preferentially accumulate in the liver and are primarily internalized by Kupffer cells, where they activate TLR4-dependent TNF production, leading to hepatic inflammation, metabolic dysfunction, and impaired drug-metabolizing capacity (Wang G. et al., 2023). These findings provide new insights into the systemic metabolic consequences of cancer and identify tumor-derived EVs, as well as downstream mediators such as TNF, as potential therapeutic targets for alleviating cachexia-associated liver dysfunction.

### 3.5 EV surface determinants of organ-specific tropism

Emerging evidence indicates that EVs display tissue- and organ-specific tropism, which is influenced, at least in part, by molecular determinants on the EV surface (Choi et al., 2024). Integrins and tetraspanins are among the membrane-associated molecules implicated in exosome recognition, adhesion, and uptake by recipient cells (Hoshino et al., 2015). Distinct EV integrin profiles have been associated with preferential accumulation in specific organs (Edelmann and Kima, 2022), suggesting that interactions between EV surface molecules and tissue-specific receptors or extracellular matrix components may contribute to organ-selective EV trafficking.

#### 3.5.1 EV surface determinants of adipose tissue targeting

Experimental evidence supports a role for EV surface molecules in adipose tissue targeting in CAC. Shibata et al. demonstrated that pancreatic cancer-derived EVs preferentially accumulated in adipose tissue and identified EV-associated integrins ITGA6 and ITGB1 as important mediators of EV-adipocyte interactions. Genetic deletion of either ITGA6 or ITGB1 reduced EV-adipocyte association, while simultaneous deletion of both integrins markedly attenuated EV-induced lipolysis (Shibata et al., 2022). These findings provide experimental evidence that specific EV surface molecules can contribute to adipose tissue tropism and may thereby facilitate tumor-derived EV-mediated adipose wasting in CAC.

#### 3.5.2 EV surface determinants of skeletal muscle targeting

Evidence for skeletal muscle targeting is emerging, although the underlying molecular mechanisms remain less well characterized. Experimental studies have demonstrated that EV surface engineering enhances their delivery to skeletal muscle. For example, Wang et al. engineered exosomes by fusing the EV membrane protein Lamp2b with a muscle-specific targeting peptide (SKTFNTHPQSTP), thereby conferring muscle-targeting properties to the engineered exosomes. In a mouse model of chronic kidney disease, these engineered exosomes enhanced miR-26a delivery to skeletal muscle and attenuated muscle wasting (Wang et al., 2019). In addition, Devan et al. demonstrated that EVs derived from different functional states of C2C12 muscle cells exhibited distinct uptake patterns, with exosomes from quiescent myoblasts showing the strongest internalization by differentiated myotubes (Devan et al., 2024).

These findings support the existence of selective EV-skeletal muscle interactions, although the molecular determinants underlying such interactions remain incompletely understood. Therefore, whether tumor-derived EVs naturally possess specific surface molecules that mediate preferential recognition, uptake, or accumulation in skeletal muscle remains largely unknown. Identifying such endogenous determinants in CAC may provide new insights into tumor-muscle communication and facilitate the development of EV-based strategies for selectively blocking pathogenic EV uptake or delivering therapeutics to skeletal muscle.

## 4 EVs from tumors: novel biomarkers for CAC

**FIGURE 3:**
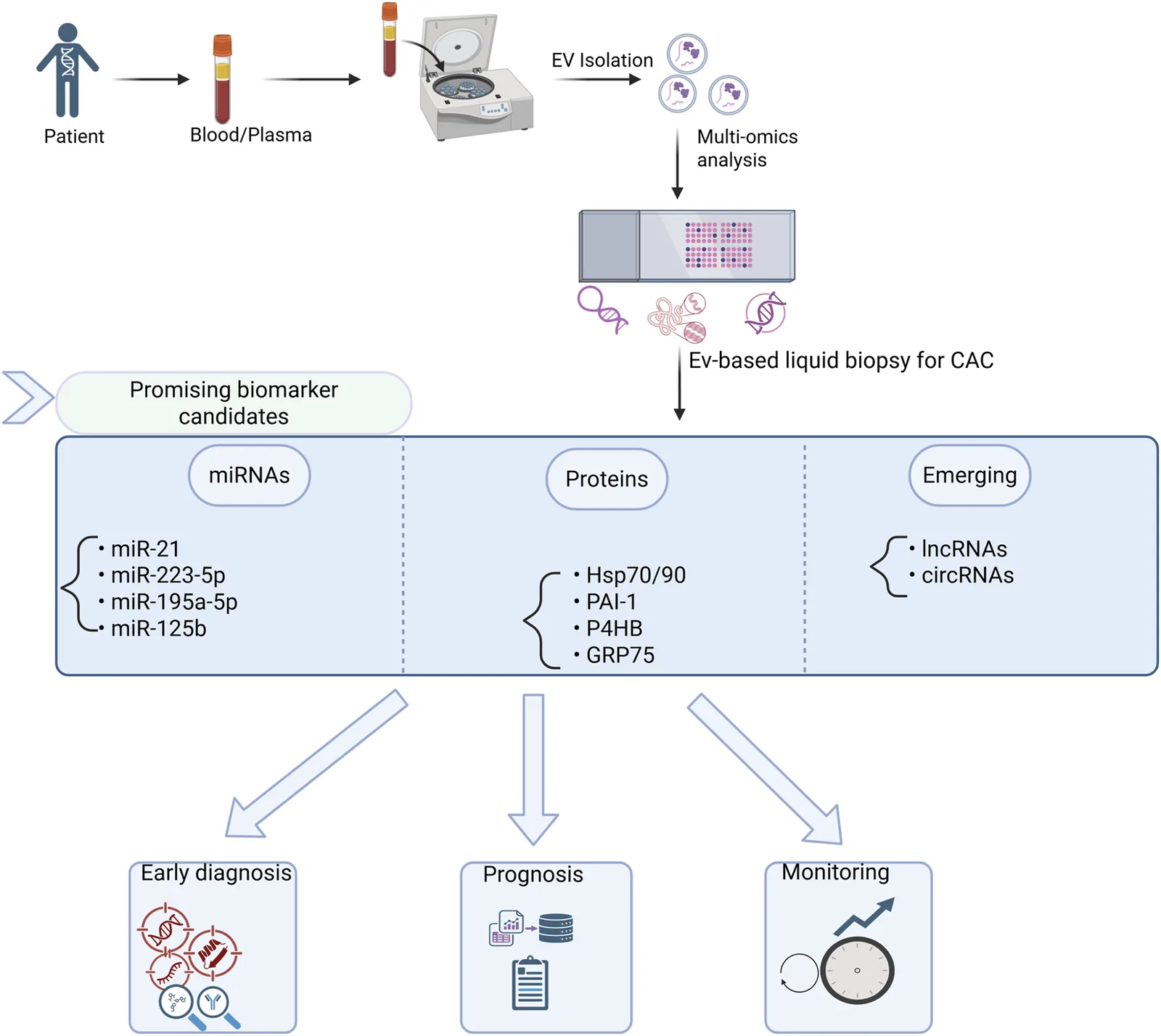
EV-based liquid biopsy strategy for CAC. EVs isolated from patient blood are subjected to multi-omic profiling to identify candidate biomarkers. Three classes of promising biomarkers are highlighted: miRNAs such as miR-21 and miR-223-5p, proteins including Hsp70/90 and GRP75, as well as emerging biomarkers like lncRNAs and circRNAs. These biomarkers hold potential for early diagnosis, prognosis prediction, and treatment monitoring in CAC. Created in BioRender. an, X. (2026) https://BioRender.com/fncair4.

Recent studies have highlighted EVs as promising non-invasive tools for identifying CAC, owing to their ability to reflect tumor dynamics and systemic metabolic alterations (Meckes et al., 2010; Schwarz et al., 2017; Zhou E. et al., 2021). Compared with conventional circulating factors, EVs provide enhanced stability and protection of molecular cargo in biofluids, enabling more reliable detection (Hamed et al., 2024; Chitti et al., 2023; Sanghvi et al., 2025). Importantly, increasing evidence indicates that EV-derived molecules are closely associated with skeletal muscle loss and clinical outcomes, highlighting their potential utility in early diagnosis and prognosis of CAC. As shown in Figure 3, EV-derived proteins and nucleic acids represent potential biomarkers for the diagnosis and prognosis of CAC.

### 4.1 ncRNA biomarkers in CAC

Among EV-derived cargoes, ncRNAs, including miRNAs, lncRNAs, and circRNAs, have attracted considerable attention as candidate biomarkers for CAC. These molecules are frequently dysregulated in tumor-derived EVs and play critical roles in intercellular communication and metabolic reprogramming during CAC progression (Sanghvi et al., 2025; Ma et al., 2025; Wang Q. et al., 2023). Notably, their expression patterns vary among different tumor types and disease stages, suggesting potential applications in disease stratification and prognosis (Zhu et al., 2021; Zhong et al., 2025; Gao et al., 2024). Further investigation in this field will facilitate the identification of reliable biomarkers for the diagnosis, prognosis, and therapeutic evaluation of CAC.

#### 4.1.1 miRNAs as biomarkers in CAC

Among ncRNAs, EV-associated miRNAs have been the most extensively studied as biomarkers for CAC (Molfino et al., 2023; van de Worp et al., 2020). Several miRNAs, including miR-1, miR-133a/b, miR-206, and miR-486, are closely associated with muscle homeostasis (Güller and Russell, 2010; Belli et al., 2021).

A recent investigation involving patients with pancreatic ductal adenocarcinoma (PDAC) has provided compelling evidence for circulating miRNAs as markers of CAC. Using deep sequencing, miR-223-5p was found to be significantly elevated in plasma-derived EVs from PDAC patients presenting with sarcopenia. Importantly, beyond mechanistic exploration, the clinical relevance of EV-miR-223-5p was validated in a large-scale cohort. Plasma EV-miR-223-5p levels were significantly inversely correlated with skeletal muscle index, and higher EV-miR-223-5p levels were associated with reduced overall survival at 3 years. ROC analysis demonstrated moderate diagnostic accuracy (AUC = 0.711) (Xu et al., 2025). These findings not only confirm the EV origin of circulating miR-223-5p but also establish its link to clinical prognosis, offering a compelling paradigm for miRNA-based liquid biopsy.

However, several limitations remain. Many studies rely on unfractionated serum samples, making it difficult to distinguish EV-derived miRNAs from non-vesicular circulating miRNAs (Belli et al., 2021; Nik and Shahidan, 2019). In addition, the mechanisms governing selective miRNA packaging into EVs remain poorly understood, limiting their translational applicability.

#### 4.1.2 lncRNA as biomarkers in CAC

In addition to miRNAs, EV-secreted lncRNAs also exhibit considerable potential as diagnostic biomarkers for CAC. LncRNAs are a class of ncRNA molecules that lack protein-coding capacity but exert crucial regulatory roles in cellular processes (Hashemi et al., 2022). These molecules are localized in both the nucleus and cytoplasm, and their dysregulated expression is closely associated with tumor initiation and progression. However, research on lncRNAs in the context of CAC is less extensive than that on miRNAs, and relevant investigations are still ongoing. Nevertheless, several compelling findings have been reported; for instance, lncRNA H19 is transferred to recipient cancer cells via exosomes, thereby promoting tumor progression. This observation highlights the potential of lncRNA H19 as a candidate biomarker and its correlation with poor clinical prognosis in CAC (Hashemi et al., 2022). Similarly, LINC00355 (Zhao et al., 2023) demonstrates promising potential as a diagnostic biomarker for CAC.

#### 4.1.3 CircRNAs as biomarkers in CAC

CircRNAs are generated through a non-canonical splicing process in which a downstream 5′donor site is covalently linked to an upstream 3′acceptor site, forming a closed-loop structure (van de Worp et al., 2020). They are widely distributed and highly abundant in various organisms and biological fluids (Zhao et al., 2024). Notably, due to the absence of 5′caps and 3′poly(A) tails, circRNAs exhibit remarkable stability and resistance to RNase-mediated degradation (Suzuki and Tsukahara, 2014).

However, current research on EV-secreted circRNAs primarily focuses on their utility as diagnostic and prognostic biomarkers for cancer. For instance, circRELL1 is downregulated in the plasma of patients with gastric cancer (GC) (Sang et al., 2022), supporting its feasibility as a dual-purpose biomarker for GC diagnosis and prognosis. Similarly, plasma circ-IARS is significantly upregulated in patients with metastatic PDAC (Li et al., 2018), which highlights its potential as a biomarker for early diagnosis and longitudinal monitoring of disease progression. Although these studies have not specifically focused on CAC, advanced-stage cancers are frequently accompanied by cachexia, suggesting a clinically relevant association. Collectively, these findings provide a rationale for exploring EV-derived circRNAs as candidate biomarkers for CAC.

### 4.2 EV-derived proteins as potential biomarkers associated with cancer pr and CAC

In addition to ncRNAs, EV-derived proteins have also been implicated in CAC pathogenesis and may serve as potential biomarkers of cachexia-associated alterations. For instance, elevated Hsp70/90 levels in circulating EVs have been associated with skeletal muscle atrophy (Zhang et al., 2017a). Beyond their value as biomarkers, some EV-associated proteins have been directly implicated in the metabolic remodeling underlying CAC. For example, pancreatic cancer-derived EV-associated AM has been shown to activate adipocyte lipolysis through MAPK-dependent HSL phosphorylation, providing mechanistic evidence linking tumor EVs to adipose tissue wasting, an early and prominent feature of CAC (Wang et al., 2022). In addition, GRP75 has recently emerged as a promising biomarker for the early diagnosis of cancer-associated cachexia. In a study tracking the temporal progression of CAC induced by esophageal squamous cell carcinoma (ESCC), serum GRP75 levels were significantly elevated during the pre-cachectic phase. This increase coincided with the onset of WAT browning and preceded detectable weight loss and skeletal muscle atrophy. Notably, serum GRP75 was inversely correlated with WAT mass in LLC tumor-bearing mice (Chen et al., 2024b), suggesting its potential as a surrogate biomarker for adipose tissue wasting. Given that WAT browning is now widely recognized as an early event in CAC pathogenesis, GRP75 may offer a broader diagnostic window compared to muscle-specific markers, enabling earlier therapeutic intervention. More recently, exosomal EIF5A derived from LLC cells was shown to directly promote adipocyte lipolysis and adipose tissue wasting in experimental models of CAC. Mechanistically, exosomal EIF5A binds GPBAR1 mRNA and enhances its translation, thereby activating cAMP/CREB signaling and promoting lipolysis. Moreover, elevated EIF5A expression was associated with poorer overall survival in patients with lung cancer, suggesting that EV-associated EIF5A may have both mechanistic and potential prognostic relevance in cancer progression, although its value as a CAC-specific biomarker remains to be established (Xiong et al., 2023).

Collectively, current evidence indicates that EV-derived proteins may serve dual roles in CAC, acting both as functional mediators of tumor-host metabolic communication and as potential biomarkers reflecting disease progression. Nevertheless, clinical validation using cachexia-specific parameters, including muscle mass, adipose tissue loss, nutritional status, and survival outcomes, remains necessary before their application as CAC biomarkers.

## 5 Potential therapeutic strategies targeting EVs to ameliorate CAC

Although a growing number of studies have explored therapeutic strategies to ameliorate CAC, the identification of effective interventions remains challenging and depends critically on a comprehensive understanding of EV-mediated mechanisms. In particular, EVs function as key mediators of intercellular communication, orchestrating complex metabolic reprogramming across multiple tissues, including skeletal muscle and adipose tissue.

### 5.1 Intervention strategies targeting EVs for ameliorating CAC

**FIGURE 4:**
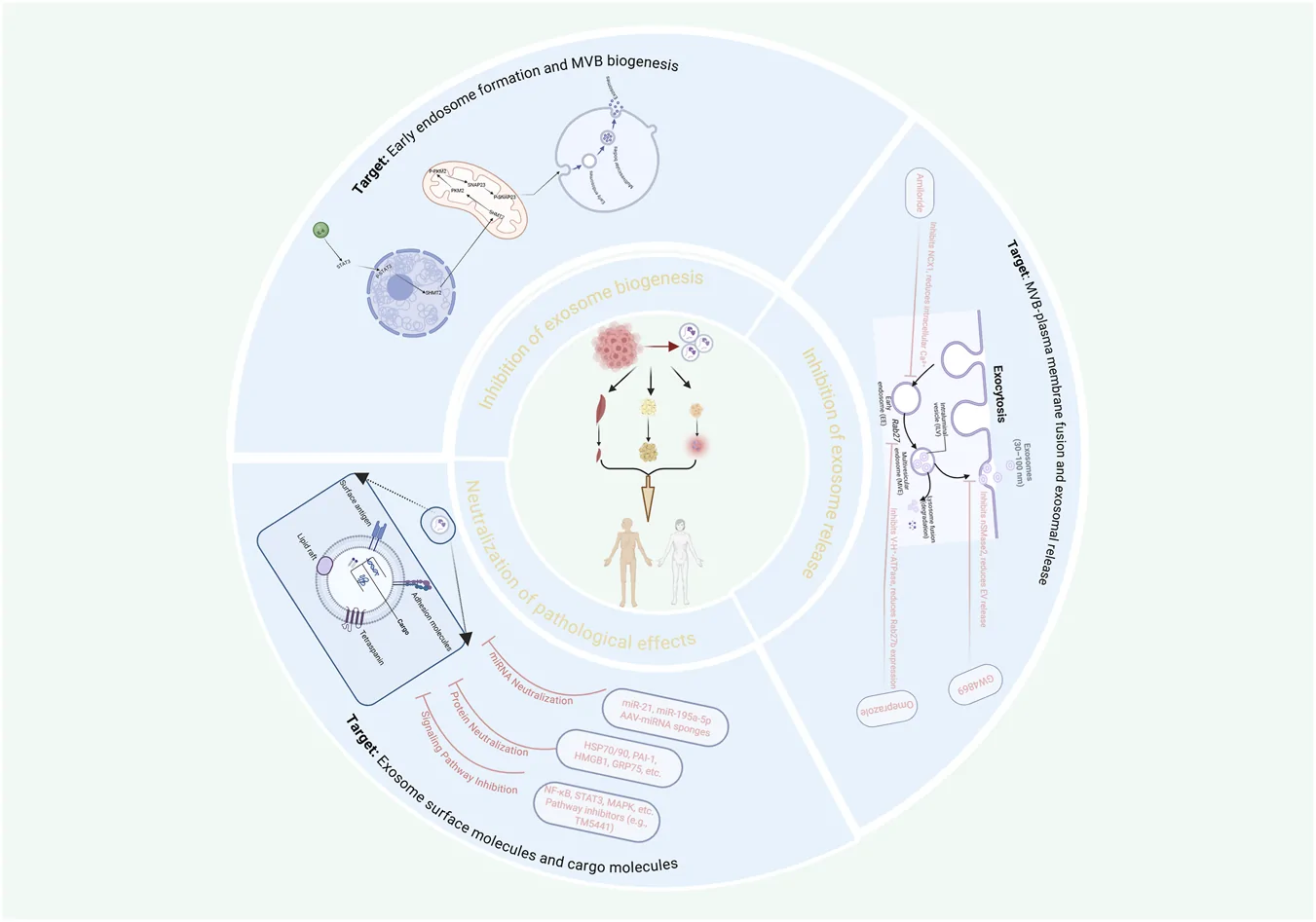
EV-targeted therapeutic strategies for CAC. Schematic illustration of the major EV-targeted intervention strategies for CAC. Therapeutic approaches include inhibition of EV biogenesis through modulation of endosomal formation and MVB biogenesis, suppression of EV release by interfering with MVB-plasma membrane fusion, and neutralization of pathogenic EVs or their cargo. Representative pharmacological inhibitors, including GW4869, omeprazole, and amiloride, together with engineered strategies targeting EV surface molecules, signaling pathways, and disease-associated cargo (e.g., miRNAs, HSP70/90, GDF15, and inflammatory mediators), are summarized. Collectively, these approaches aim to interrupt EV-mediated intercellular communication, attenuate metabolic dysfunction and tissue wasting, and improve therapeutic outcomes in CAC. Created in BioRender. an, X. (2026), https://BioRender.com/rvktktn.

In CAC, interventions targeting distinct stages of EV biology may represent an effective approach to mitigate disease progression. This section outlines potential strategies that focus on reducing the generation of pathogenic EVs by inhibiting EV biogenesis, limiting their systemic dissemination by blocking EV release, and attenuating their deleterious effects on muscle and adipose tissue through neutralization of pathogenic EV cargo. These three strategies are schematically illustrated in Figure 4.

#### 5.1.1 Traditional Chinese medicine-based modulation of EV production

EVs originate from the endosomal sorting pathway and multivesicular bodies (MVBs). (van Niel et al., 2018; Suárez et al., 2021). Targeting key enzymes or signaling pathways in this process can reduce the production of pro-cachectic EVs in tumor cells. For example, a bioactive component derived from traditional Chinese medicine (TCM), Atractylenolide I (AI), can reduce aerobic glycolysis in C26 colon cancer cells by inhibiting the STAT3/PKM2/SNAP23 signaling pathway, ultimately decreasing exosome production and IL-6 secretion (Fan et al., 2022b). Specifically, IL-6 binds to the glycoprotein 130 (gp130), activating JAK/STAT3 signaling, which further phosphorylates STAT3 and modulates downstream signaling mediators (Johnson et al., 2018). This finding clarifies the potential mechanism underlying the multi-targeted therapeutic effects of TCM-derived bioactive monomers in CAC. Furthermore, this study provides a theoretical framework for the development of modern therapeutics derived from TCM capable of modulating systemic disease networks to alleviate systemic inflammatory disorders.

However, several challenges remain before this therapeutic strategy can be translated into clinical use. Given the substantial heterogeneity in EV cargo profiles and cytokine signatures among different tumor types, whether AI exerts similar therapeutic effects across other malignancies remains uncertain. Overall, targeting EV production represents a promising strategy to suppress EV-mediated pro-cachectic signaling. Nevertheless, whether sufficient EV specificity can be achieved without disrupting the physiological secretion and functions of EVs remains an unresolved challenge.

#### 5.1.2 Inhibition of EV release

Another alternative strategy is to prevent EV release into the extracellular space. EV release depends on the fusion of MVBs with the plasma membrane (Bebelman et al., 2020). Targeting key molecules in this process can block the extracellular transmission of pro-cachectic signals. For instance, amiloride inhibits Na^+^/Ca^2+^ exchanger 1 (NCX1), thereby reducing intracellular Ca^2+^ concentrations. This reduction impairs the exocytic release of MVBs, a critical step in EV secretion (Zhou L. et al., 2021). Beyond amiloride, in the aforementioned studies investigating EVs derived from colorectal cancer cells, GW4869, a selective inhibitor of neutral sphingomyelinase 2 (nSMase2), inhibits the biogenesis and release of tumor-derived Evs, thereby suppressing lipolysis and WAT browning (Ru et al., 2024; Hu et al., 2018; Kosaka et al., 2010). Experimental studies further support this approach. In tumor-bearing mice, GW4869 administration alleviated tibialis anterior (TA) muscle atrophy and improved grip strength without significantly affecting tumor progression. Consistently, pretreatment of C26 cancer cells with GW4869 abolished the atrophic effects of conditioned medium on C2C12 myotubes (Miao et al., 2021; Chen et al., 2019). In addition, Rab GTPases are key regulators of EV release. Rab27b, which is highly expressed in several tumor types, mediates docking of MVBs to the plasma membrane. In pancreatic cancer models, ZIP4 upregulates Rab27b via CREB activation, thereby enhancing EV release and promoting cachexia. Knockdown of ZIP4 reduces EV secretion and alleviates muscle atrophy (Yang et al., 2019; Ostrowski et al., 2010).

Furthermore, omeprazole diminished EV release by inhibiting V-H^+^-ATPase activity in a dose-dependent manner, thereby increasing lysosomal pH and impairing MVB maturation. Moreover, it directly downregulates Rab27b mRNA and protein expression in LLC lung cancer cells (Liu Z. et al., 2022; Hendrix et al., 2013). These approaches highlight the therapeutic potential of inhibiting EV release as a means of reducing systemic dissemination of pro-cachectic signals. Nevertheless, given the essential roles of EVs in normal physiological processes, long-term inhibition may lead to unintended side effects.

#### 5.1.3 Neutralization of pathological effects

Another strategy focuses on neutralizing the pathological effects induced by EV-derived cargo molecules. In the lung carcinoma cachexia model, neutralization of extracellular HSP70/90 inhibited activation of the autophagy-lysosome pathway and alleviated skeletal muscle atrophy (Liu Z. et al., 2022; Zhang et al., 2017b). Similarly, studies have demonstrated that glycyrrhizin, an inhibitor of high mobility group box 1 (HMGB1), counteracts exosome-derived HMGB1-induced muscle protein degradation by inhibiting the activation of the TLR4/NF-κB pathway (Li L. et al., 2021). In addition, for muscle atrophy driven by exosomal PAI-1, the PAI-1 inhibitor TM5441 effectively blocks the downstream STAT3 signaling pathway activated by exosomal PAI-1, thereby alleviating muscle atrophy (Shin et al., 2022). Overall, targeted blockade of EV-derived cargo may alleviate CAC progression.

Of note, in addition to neutralizing known pro-catabolic proteins, targeting specific miRNAs carried by tumor-derived EVs has emerged as a promising therapeutic strategy. In PDAC-associated cachexia, miR-223-5p was identified as a critical mediator of muscle atrophy. Silencing miR-223-5p using either synthetic inhibitors *in vitro* or an AAV-delivered miRNA sponge *in vivo* effectively attenuated muscle loss and improved grip strength in mouse models (Xu et al., 2025). These findings define a regulatory axis involving miR-223-5p, MAFA, METTL14, and m6A methylation, offering potential targets for precision intervention in PDAC cachexia. A key contribution of this study is its integration of EV-miRNA biology with transcriptional regulation and RNA epitranscriptomics. It demonstrates that tumor-derived EVs can induce muscle wasting not only by activating established proteolytic pathways but also by reprogramming muscle metabolism at the level of RNA methylation.

## 6 Current landscape and limitations of pharmacological interventions

Given the complex pathogenesis of CAC, no single therapeutic agent has demonstrated sufficient efficacy; instead, emerging pharmacological strategies - including novel inhibitors, repurposed drugs, and integrated multimodal approaches - offer potential avenues for managing this devastating syndrome.

### 6.1 Development of novel drugs and active compounds

In light of the role of EVs in CAC pathogenesis, various bioactive small molecules have been developed and isolated, offering potential benefits for improving the quality of life of CAC patients. The current landscape of novel drug development and active monomers for CAC is presented in Table 3.

**TABLE 3:** Current landscape of novel drug development and active monomers for CAC.

Agent	Mechanism of action	Clinical stage	Primary indications	Major adverse effects	References
Atractylenolide I	Inhibits IL-6 biosynthesis and suppresses the production of tumor-derived extracellular vesicles	Randomized controlled exploratory clinical trial	CAC	Not reported	Fan et al. (2022b)
Withanone	Acts as a specific GRP75 inhibitor to block the formation of the GRP75-ANT2-UCP1 complex, thereby preventing WAT browning	Preclinical	CAC (ESCC models)	Not applicable (preclinical stage)	Chen et al. (2024c)
Ponsegromab	Functions as a GDF-15 neutralizing antibody that blocks the interaction between GDF-15 and the GFRAL receptor	Phase 2	CAC associated with non-small cell lung cancer, pancreatic cancer, and colorectal cancer	Diarrhea, nausea, and vomiting	Groarke et al. (2024)
Paeoniflorin	Attenuates muscle wasting by inhibiting the TLR4/NF-κB signaling pathway and activating the AKT/mTOR signaling cascade	Preclinical	Muscle atrophy induced by CAC	Not applicable (preclinical stage)	Zhu et al. (2024)
Ursolic acid	Alleviates muscle wasting through STAT3 pathway inhibition while exhibiting antioxidant and anti-inflammatory properties	Preclinical	Muscle atrophy induced by CAC	Not applicable (preclinical stage)	Chen et al. (2024c)
Baicalein	Modulates the AKT signaling pathway to inhibit muscle protein degradation	Early clinical exploration	Muscle atrophy induced by CAC	Not reported	Song et al. (2024)
GinsenosideRd	Binds to STAT3, inhibiting its phosphorylation and nuclear translocation. This suppresses the expression of atrophy-related genes such as atrogin-1, MuRF-1, and MSTN.	Preclinical	Muscle atrophy induced by CAC	Not applicable (preclinical stage)	Wijaya et al. (2022)
Gintonin	Activates the LPAR/Gαi2 pathway, reducing oxidative stress and inflammation. It downregulates atrogin-1 and MuRF-1, thereby inhibiting muscle atrophy	Preclinical	Lung cancer-related muscle wasting	Not applicable (preclinical stage)	Wijaya et al. (2021)

#### 6.1.1 Atractylenolide I (AI)

Among these, AI-based approaches have shown promise (Fan et al., 2022b). However, the marked heterogeneity of EV profiles and cytokine signatures across different tumor types poses a significant barrier to their broad applicability. Future investigations should aim to establish a causal link between the inhibition of EV biogenesis and the amelioration of CAC.

#### 6.1.2 Withanone

Withanone, a selective inhibitor of GRP75, represents a novel strategy targeting mitochondrial metabolic regulation. In an esophageal squamous cell carcinoma model, administration of Withanone during the pre-cachectic stage significantly suppressed WAT browning, reduced UCP1 expression, and preserved body weight. Notably, combination therapy with Withanone and cisplatin not only inhibited tumor growth but also attenuated chemotherapy-associated cachexia (Chen et al., 2024b). However, given the essential role of GRP75 as a mitochondrial chaperone, systemic inhibition may result in unintended metabolic consequences in normal tissues. Therefore, its long-term safety and applicability across different cancer types require further investigation.

#### 6.1.3 Ponsegromab

Ponsegromab, a monoclonal antibody targeting GDF-15, represents a promising advancement in CAC therapy. A phase II clinical trial reported that patients receiving 400 mg of Ponsegromab achieved a median body weight gain of 2.81 kg over 12 weeks, accompanied by improvements in appetite and physical performance (Groarke et al., 2024). Following the positive results from the Phase II trial, a global multicenter Phase IIb/III study (NCT06989437) was initiated in 2025. This study is designed to evaluate the efficacy and safety of Ponsegromab in combination with first-line chemotherapy. Nevertheless, certain limitations remain. The efficacy of Ponsegromab was observed primarily in patients with elevated GDF-15 levels ≥1500 pg/mL or higher. Additionally, the follow-up duration was only 12 weeks, leaving long-term survival benefits yet to be confirmed. Overall, the development of bioactive monomeric agents has shown considerable promise in improving the quality of life in patients with CAC.

### 6.2 Drug repurposing strategies

In addition to the development of novel bioactive monomers, research efforts have increasingly focused on drug repurposing as a therapeutic strategy. Repurposed drugs offer several distinct advantages in the treatment of CAC, including well-characterized safety and pharmacokinetic profiles, the ability to initiate clinical trials without prolonged preclinical development, and notable cost-effectiveness. The repurposed pharmaceuticals for the management of CAC are presented in Table 4.

**TABLE 4:** Repurposed pharmaceuticals for the management of CAC: mechanisms and clinical status.

Agent	Original therapeutic class	Mechanism of action	Clinical stage	Primary indications in cachexia	Reported adverse events	References
Metformin	Biguanide Antidiabetic Agent	Activates AMPK and inhibits the mTOR pathway; induces autophagy	Preclinical	Cachexia associated with pancreatic, colorectal, and breast cancers	Gastrointestinal disturbances including diarrhea and nausea; risk of lactic acidosis	Oliveira and Gomes-Marcondes (2016)
Hydroxychloroquine	Antimalarial and Immunomodulatory Agents	Inhibits autophagy; promotes secretion of the tumor suppressor Par-4	Phase 1	Potential amelioration of cachexia-induced muscle wasting	Not specified in current data	Wang et al. (2018)
Amiloride	Potassium-Sparing Diuretic	Inhibits the NCX1 to reduce intracellular Ca^2+^ concentration	Preclinical	Cachexia associated with colorectal and lung cancers	Electrolyte Imbalance	Zhou et al. (2021b)
Omeprazole	Proton Pump Inhibitor	Inhibits V-H^+^-ATPase to elevate lysosomal pH and interfere with multivesicular body maturation; downregulates Rab27b expression	Preclinical	Cachexia associated with pancreatic and gastric cancers	Gastrointestinal adverse effects including nausea and vomiting	Liu et al. (2022b)

#### 6.2.1 Metformin

Metformin, a first-line therapy for type 2 diabetes, has gained attention for its potential anti-cachectic effects. Mechanistically, metformin activates AMPK by inhibiting mitochondrial respiratory chain complex I, thereby modulating cellular energy metabolism (Oliveira and Gomes-Marcondes, 2016). However, its effects on EV biology appear to be context-dependent. In an endometrial cancer model, metformin suppressed EV secretion and reduced adiposity and tumor growth (Sakaue et al., 2024). In contrast, in ovarian cancer, metformin increased exosome biogenesis and release, as evidenced by upregulation of CD63, Alix, and Rab27b (Abbasi et al., 2024). This discrepancy may be attributed to tumor type specificity, differences in the metabolic microenvironment, or the distinct cellular sources of EVs.

#### 6.2.2 Hydroxychloroquine

Hydroxychloroquine, a drug commonly used in autoimmune diseases, inhibits autophagosome–lysosome fusion (Mauthe et al., 2018). Given that IL-6-mediated signaling promotes muscle atrophy partly through autophagy activation, hydroxychloroquine may exert protective effects in CAC (Pettersen et al., 2017). Moreover, as observed in COVID-19 and connective tissue diseases, CAC is characterized by elevated levels of inflammatory cytokines, and chloroquine or hydroxychloroquine has demonstrated immunomodulatory potential in these conditions. Nevertheless, long-term use of these agents is associated with retinal toxicity and an increased risk of age-related macular degeneration (Melles and Marmor, 2014). Their use should therefore be considered only in refractory CAC cases, with short-term, low-dose administration and close ophthalmologic monitoring.

#### 6.2.3 Omeprazole and amiloride

Similarly, omeprazole and amiloride represent complementary repurposing strategies that block EV release through distinct mechanisms (Zhou L. et al., 2021; Liu Z. et al., 2022). However, their application warrants careful risk-benefit evaluation. Amiloride, a diuretic, may exert anti-cachexia effects at doses approaching those used for diuresis, thereby posing a risk of electrolyte imbalance. Although repurposing omeprazole offers the advantage of immediate clinical translatability, key considerations include the route of administration and the need for sustained drug exposure. Future clinical trials should explore alternative delivery strategies, such as subcutaneous administration or sustained-release formulations.

### 6.3 Multimodal combination strategies

Therapeutic approaches for CAC are increasingly shifting from single-target interventions to multimodal strategies that integrate pharmacological and non-pharmacological components. Emerging evidence suggests that targeting EV-related processes in conjunction with multidisciplinary interventions may provide a more integrated therapeutic framework for CAC. The current and potential therapeutic agents for the management of CAC-related symptoms are presented in Table 5.

**TABLE 5:** Current and potential therapeutic agents for the management of CAC-related symptoms.

Drug class	Representative agent	Mechanism of action	Clinical stage	Primary indications	Major adverse effects	References
Ghrelin Receptor Agonists	Anamorelin	Mimics endogenous ghrelin to stimulate appetite and growth hormone secretion, promoting increases in both lean body mass and fat mass	Approved in Japan, South Korea, and other Asian countries for cachexia	Cancer anorexia cachexia syndrome (CACS) associated with non-small cell lung cancer, gastric cancer, pancreatic cancer, and colorectal cancer	Nausea, vomiting, diarrhea, constipation, hyperglycemia, and other gastrointestinal symptoms	Temel et al. (2016); Katakami et al. (2018)
Selective Androgen Receptor Modulators (SARMs)	Enobosarm	Selectively activates androgen receptors in skeletal muscle and bone to promote muscle growth and strength enhancement	phase II	Muscle wasting and functional decline CAC	Potential hepatotoxicity, testosterone suppression in males, and hirsutism in females	Dobs et al. (2013); Dalton et al. (2011); Bedi et al. (2021)
​	Espindolol	Acts as a beta-blocker and 5-HT1A receptor agonist, potentially reducing energy expenditure and improving muscle synthesis	Phase III	CAC	Hypotension, bradycardia, dizziness, fatigue, and dyspepsia	Stewart et al. (2016)
Appetite Stimulants/Progestins	Megestrol Acetate	Synthetic progestin that primarily promotes weight gain by increasing appetite	Commonly used in palliative care settings; primarily increases fat mass rather than lean muscle mass	Cancer-induced anorexia and weight loss	Thromboembolic events, edema, hyperglycemia, and adrenal suppression	Currow et al. (2021)
Anti-inflammatory Agents	dexamethasone	Exerts broad anti-inflammatory effects by suppressing the release of pro-inflammatory cytokines	Used clinically for short-term or palliative management	Relief of inflammation and improvement of appetite	Long-term use may lead to muscle catabolism, immunosuppression, hyperglycemia, edema, and osteoporosis	Currow et al. (2021)
​	Anti-IL-6 Antibodies (e.g., Siltuximab)	Blocks the IL-6 signaling pathway to attenuate inflammatory responses	Under clinical investigation	Cancer cachexia, particularly in patients with elevated IL-6 levels	Increased risk of infection and infusion-related reactions	Angevin et al. (2014)
Nutritional Supplements/Modulators	Eicosapentaenoic Acid (EPA)/Omega-3 Fatty Acids	Possesses anti-inflammatory properties that may help ameliorate cachexia, particularly in patients with gastrointestinal tumors	Used as a nutritional supplement	Cachexia associated with gastrointestinal tumors	Gastrointestinal discomfort and increased bleeding risk at high doses	Sánchez-Lara et al. (2014)

#### 6.3.1 Multimodal interventions and stratified treatment strategies

**FIGURE 5:**
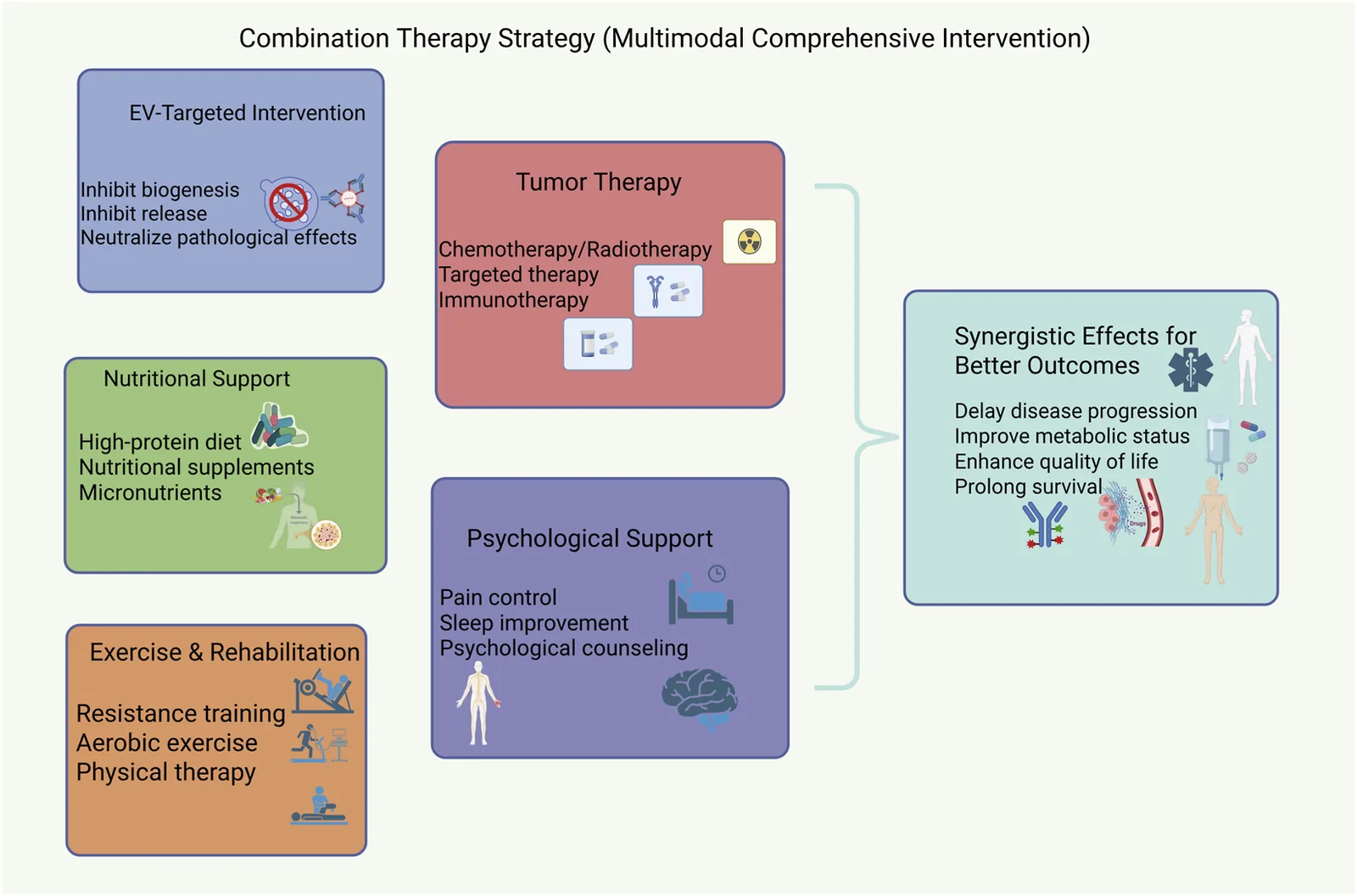
Integration of EV-targeted therapy into multimodal management of CAC Proposed comprehensive intervention strategy integrating EV-targeted therapy with current standard-of-care approaches for CAC. EV-directed interventions, including inhibition of EV biogenesis, suppression of EV release, and neutralization of pathogenic EVs, may complement conventional anticancer therapies (chemotherapy, radiotherapy, targeted therapy, and immunotherapy). Nutritional support, exercise rehabilitation, and psychological care further contribute to preserving skeletal muscle mass, improving metabolic homeostasis, and enhancing patient wellbeing. The synergistic integration of these interventions may delay cachexia progression, improve quality of life, enhance treatment response, and prolong survival. Created in BioRender. an, X. (2026), https://BioRender.com/rvktktn.

The multifactorial nature of CAC limits the effectiveness of monotherapies, supporting the adoption of integrated treatment strategies. Biomarkers, including circulating EV levels, may help guide therapeutic decision-making and optimize treatment responses. The integration of EV-targeted therapies into multimodal management of CAC is presented in Figure 5.

Clinical guidelines emphasize the importance of individualized multidisciplinary management, incorporating nutritional support, pharmacological interventions, and exercise-based rehabilitation (Soria Rivas et al., 2024). The results of the MENAC international multicenter trial indicate that multimodal interventions achieve weight stabilization, thereby providing initial support for a combined therapeutic framework (Solheim et al., 2017). However, the observed benefits were limited to weight maintenance, suggesting that current integrated approaches do not fully address the key pathogenic drivers of the disease. In light of these limitations, future clinical strategies may benefit from adopting stage-specific and adaptive treatment frameworks. During the pre-cachexia phase, a combination of EV inhibitors such as high-dose omeprazole, nutritional support, and an exercise prescription could be implemented to prevent muscle loss. In the cachexia phase, metabolic modulators such as Ponsegromab, neutralizing antibodies targeting heat shock proteins, and anti-inflammatory therapy could be applied. For refractory cachexia, palliative symptom management combined with psychosocial support and home-based nutritional care may be appropriate.

#### 6.3.2 Targeting the primary tumor remains essential

Within this multilayered therapeutic framework, sustained control of the primary tumor remains a fundamental prerequisite. CAC is not an independent disorder but rather a tumor-driven systemic metabolic condition, and its severity is closely linked to tumor burden. Accordingly, treatment directed at the underlying malignancy constitutes an essential component of CAC management. In studies of pancreatic CAC, simultaneous suppression of tumor progression and mitigation of metabolic wasting is critical for improving overall outcomes.

Taken together, CAC should be viewed as a dynamic, tumor-driven systemic process rather than an isolated metabolic endpoint. This perspective underscores the need to move beyond single-pathway interventions toward integrated therapeutic strategies. Although mechanistic studies remain essential for elucidating disease biology, their translational impact may be limited unless integrated with effective treatment of the primary tumor. Future research should therefore prioritize the development of comprehensive approaches that combine effective tumor control with interventions targeting tumor-induced metabolic disturbances, thereby establishing a more clinically translatable therapeutic framework.

## 7 Challenges and future perspectives of EVs in CAC

Despite substantial progress, four major barriers continue to hinder clinical translation of EV-based applications in CAC: (i) biomarker validation, (ii) EV isolation and characterization, (iii) biological heterogeneity, and (iv) therapeutic delivery.

### 7.1 Challenges and future perspectives of EV-based biomarkers for CAC

The intrinsic stability of EVs and their enrichment with tumor-associated proteins, lipids, and non-coding RNAs have positioned them as promising biomarkers for the early diagnosis, prognosis, and disease monitoring of CAC (Semeradtova et al., 2025). Compared with conventional circulating biomarkers, EV cargoes are better protected from enzymatic degradation and more accurately reflect the molecular characteristics of their parental cells. Consequently, EV-derived molecules such as miR-21 have demonstrated considerable potential for non-invasive liquid biopsy. Despite these advantages, several obstacles hinder their clinical application. Current EV isolation methods remain insufficiently standardized, making it difficult to obtain highly pure and biologically intact EV populations across laboratories. Moreover, many studies rely on unfractionated serum- or plasma-derived EVs, preventing definitive identification of the cellular origin of candidate biomarkers (Zhang et al., 2017a; Herreros-Villanueva and Bujanda, 2016). As a result, the specificity and reproducibility of several reported EV-associated biomarkers remain uncertain. Future investigations should therefore prioritize standardized isolation protocols, validation of EV cellular origin, integrated multi-omics characterization, and multicenter prospective clinical studies to establish robust EV-based biomarker panels for CAC.

### 7.2 Limitations and future directions of EV isolation and characterization

Reliable EV isolation is fundamental for both mechanistic studies and clinical translation. However, no currently available isolation strategy can simultaneously achieve high purity, high recovery, preservation of vesicle integrity, and scalability for routine clinical use (Tutanov et al., 2025). Each method has inherent trade-offs, and no single approach currently meets all clinical requirements.

- Differential ultracentrifugation is widely used and suitable for large sample volumes, but contamination with abundant proteins and lipoproteins may compromise downstream analyses (Shu et al., 2020; Visan et al., 2022).
- Density gradient centrifugation improves purity but may not efficiently recover EVs of similar sizes, such as microvesicles (de Menezes-Neto et al., 2015).
- Polymer-based precipitation offers high throughput but can introduce non-specific co-precipitates that confound quantitative and omics analyses (Jong et al., 2017).
- Immunoaffinity capture allows selective enrichment based on surface markers, but scalability and cost remain limiting factors (Zhao et al., 2016).
- Microfluidic technologies provide integration, low sample consumption, and rapid enrichment, but high fabrication costs and limited large-scale production capacity constrain widespread adoption (Zhang et al., 2019; Hassanpour et al., 2021).

Future technological advances should focus on combining complementary isolation strategies with advanced characterization techniques, including single-vesicle analysis and multi-omics profiling, to improve EV purity while preserving biological activity. Such integrated approaches will facilitate more reproducible functional studies and accelerate the clinical translation of EV research.

### 7.3 Limitations and prospects of EV applications

Beyond their diagnostic potential, EVs have emerged as attractive therapeutic targets and endogenous nanocarriers for drug delivery. Therapeutic strategies targeting EV biogenesis, cargo loading, secretion, or cellular uptake may interrupt tumor-host intercellular communication. Furthermore, engineered EVs exhibit favorable biocompatibility, low immunogenicity, and intrinsic tissue-targeting properties, making them promising platforms for precision drug delivery.

However, substantial barriers remain before EV-based therapies can be translated into clinical practice. Most available evidence is derived from cell culture systems and animal models, whereas high-quality clinical studies remain scarce. In addition, EV heterogeneity, incomplete understanding of selective cargo loading mechanisms, limited tissue-specific targeting efficiency, potential off-target effects, and the absence of standardized manufacturing and quality-control procedures continue to restrict clinical development.

Collectively, current evidence suggests that EVs should be regarded not merely as carriers of biomolecules but as dynamic mediators of tumor-host intercellular communication throughout the development of CAC. By integrating metabolic remodeling, inflammatory signaling, and multi-organ crosstalk, EVs provide a unifying framework for understanding the systemic pathogenesis of CAC and represent promising targets for future precision diagnostic and therapeutic strategies.

## 8 Conclusion

The accumulating evidence summarized in this review demonstrates that EVs are not merely passive carriers of biomolecules but active mediators of intercellular communication throughout CAC progression. Importantly, the unique biological properties of EVs provide promising opportunities for clinical translation. Nevertheless, significant challenges remain, including EV heterogeneity, incomplete understanding of cargo-sorting mechanisms, the lack of standardized isolation and characterization protocols, and insufficient clinical validation.

Future research should therefore focus on integrating advanced EV isolation technologies, single-vesicle and multi-omics analyses, *in vivo* tracking approaches, and well-designed clinical studies to elucidate the mechanisms underlying EV-mediated communication and facilitate clinical translation. A deeper understanding of EV biology will not only advance our knowledge of CAC pathogenesis but also promote the development of innovative diagnostic biomarkers and therapeutic strategies, ultimately improving the management and quality of life of patients with CAC. Collectively, the evidence reviewed here supports EV-mediated tumor-host communication as a unifying framework for understanding the systemic pathogenesis of CAC.

Nevertheless, several important questions remain unanswered and warrant further investigation:

- What molecular mechanisms govern the organotropism of EVs?
- What drives the heterogeneity of EV cargo among different cancer types?
- How is tissue specificity established in EV-mediated targeting of adipose tissue versus skeletal muscle?
- Can tumor-derived EVs directly traverse the blood-brain barrier (BBB), or are their effects on the CNS mediated through indirect peripheral signaling pathways?

## Glossary

AM: Adrenomedullin
BAT: Brown adipose tissue
CAC: Cancer-associated cachexia
CNS: Central nervous system
CREB: CAMP response element-binding protein
ERS: Endoplasmic reticulum stress
ESCC: Esophageal squamous cell carcinoma
EVs: Extracellular vesicles
GC: Gastric cancer
GDF-15: growth differentiation factor 15
GPC-1: Glypican-1
GRP75: glucose-regulated protein 75
HMGB1: High mobility group box 1
HSL: Hormone-sensitive lipase
JAK: Janus kinase
MAPK: Mitogen-activated protein kinase
MSTN: Myostatin
MVBs: Multivesicular bodies
NF-κB: Nuclear factor kappa-B
NCX1: Na^+^/Ca^2+^ exchanger 1
nSMase2: Neutral sphingomyelinase 2
OSCC: Oral squamous cell carcinoma
PAI-1: Plasminogen activator inhibitor-1
PDAC: Pancreatic ductal adenocarcinoma
P4HB: Prolyl 4-hydroxylase subunit beta
STAT3: Signal transducer and activator of transcription 3
TA: Tibialis anterior
TCM: Traditional Chinese medicine
TLR2: Toll-like receptor 2
UCPs: Uncoupling proteins
UPS: Ubiquitin-proteasome system
WAT: White adipose tissue
